# Orbital metastatic neuroblastoma presenting as posttraumatic exophthalmos

**Published:** 2020

**Authors:** Ana Maria Frunză, Ovidiu Samoilă

**Affiliations:** * Ophthalmology Clinic, Cluj-Napoca, Cluj; ** “Iuliu Hațieganu” University of Medicine and Pharmacy, Cluj-Napoca, Cluj

**Keywords:** neuroblastoma, orbit, exophthalmia, trauma

## Abstract

A 2-year-old female patient with a recent history of head trauma was admitted to the Ophthalmology Clinic with left exophthalmos. A differential diagnosis between traumatic and tumoral etiology was made. The orbitocranial MRI and fine needle ganglion biopsy settled the malignant etiology of the exophthalmia. Further investigations at the Pediatric Oncology Clinic decided on the diagnosis of orbital metastatic neuroblastoma. This case report presented an unusual association: orbital metastatic neuroblastoma becoming clinically positive soon after a head trauma.

## Introduction

Orbital neuroblastoma metastasis is the second most prevalent malignant orbital tumor, after rhabdomyosarcoma [**1**]. Neuroblastoma is a malignant tumor of sympathetic paravertebral ganglia that typically affects very young children and it is the most common malignancy of infancy [**2**]. Usually, orbital neuroblastoma presents with acute exophthalmos, mimicking a traumatic etiology: periorbital ecchymosis, the “raccoon eyes” due to intratumoral spontaneous necrosis [**1**].

## Case Report

A 2-year-old female patient was admitted to the Ophthalmology Clinic with left exophthalmos. 

One month earlier, she had suffered a minor head trauma, with frontal hematoma. Two weeks following that episode, she presented a progressive left exophthalmia. She had no significant other medical or family history.

The clinical examination revealed bilateral normal visual acuity, normal pupillary reflexes, left exophthalmos, displaced downward, with limited abduction and elevation movements, with exophthalmometry measurements of 7 mm on right and 14 mm on the left side and a firm frontotemporal profoundly adherent tumoral mass without skin modifications. A supraclavicular firm adherent nodule was also found by palpation. 

Biomicroscopic examination of the fundus showed a deformed upper half of the posterior pole without any obvious mass inside the globe.

**Fig. 1:**
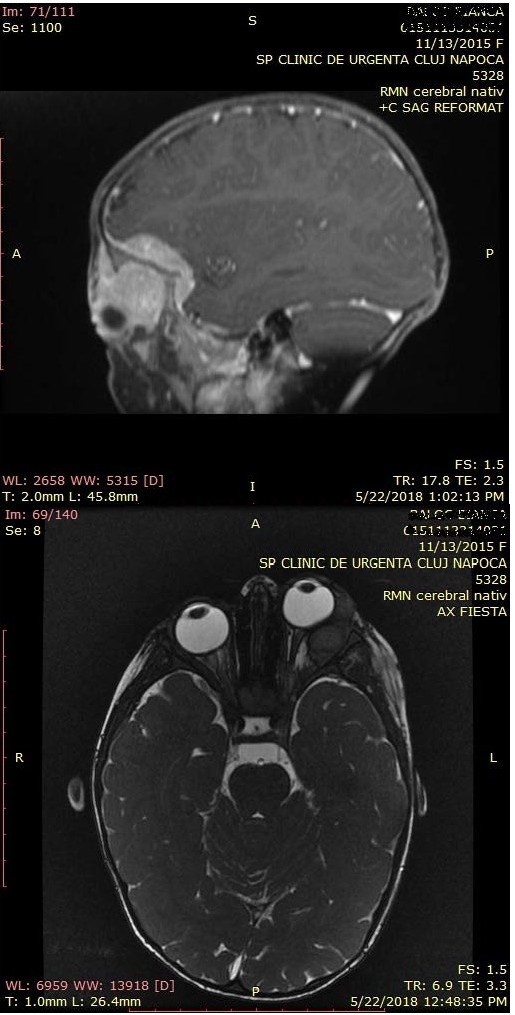
MRI of the head showing a polypoid orbital tumoral mass with extension into the left frontal, temporal, sphenoid part, into the left temporal muscle and the left frontal conjunctive tissue

The biochemical blood analysis revealed a mild anemia and a mild inflammatory syndrome. The echography of the left eye and orbital-head CT were equivocal and traumatic etiology could not be ruled out. A head MRI was performed and revealed an intra and extraconal tumoral mass with endocranial extension and enhanced contrast uptake (**Fig. 1**).

**Fig. 2:**
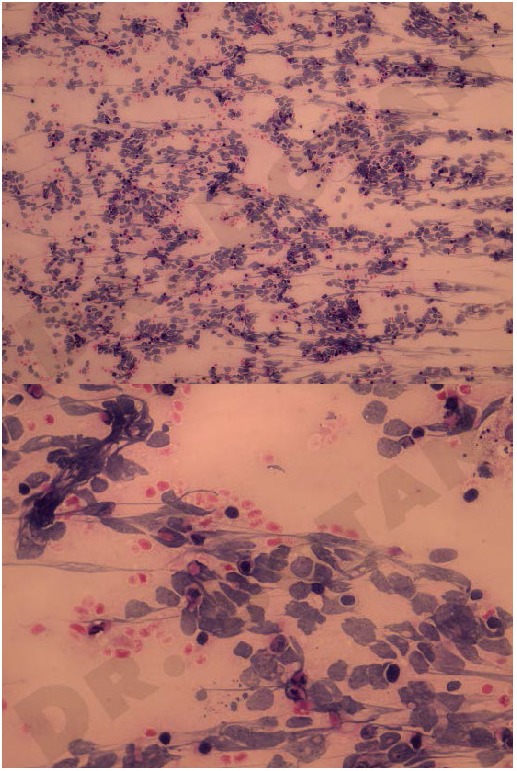
Fine needle aspiration biopsy, suggestive of a malignant tumor, small blue cells

We consulted the Pediatric Oncology Department and they recommended the biopsy from the orbital tumor, which was negative, and a fine needle aspiration of the left supraclavicular adenopathy, which was suggestive of a malignant tumor with small blue cells (**Fig. 2**).

At that moment, the patient was transferred to the Pediatric Oncology Department. The highly positive Enolase Neuron Specific marker from the blood sample, highly positive metanephrines and the abdominal echography and thoracic CT revealed a gigantic abdominal-thoracic tumoral mass and oriented towards the positive diagnostic of abdominal-thoracic neuroblastoma with orbital and ganglia metastasis.

**Outcome and Follow-up**

The patient underwent one course of chemotherapy: Vincristine, Cyclophosphamide and Doxorubicin, but parents refused further treatment courses, and the patient died at 3 months after presentation.

## Discussion

Differential diagnosis in our case included neoplasms, inflammations, and trauma. 

Neurologic intracranial tumors could manifest after minor head trauma, due to rupture of intra-tumoral dilated vessels [**3**,**4**]. Cases of orbital subperiosteal hematomas after trauma were also reported, without acute signs of orbital engagement and with later exophthalmos [**5**]. However, a case of orbital neuroblastoma, becoming clinically manifest after minor head injury, has not been reported previously. Subperiosteal hematoma was the principal differential diagnosis in our case. 

The tumor mimicked a hematoma on CT and the radiologist was able to pinpoint the diagnosis only on MRI.

The International Neuroblastoma Risk Group (INRG) utilizes a classification system that takes into account the imaging stage, age on onset and pathology (histology, differentiation) [**6**]. Localized tumors and age below 18 months at the moment of diagnosis carry the best prognosis and low risk. Our case had a metastatic disease in the orbit, age of 24 months at diagnosis, and high risk. High age children have the worst prognosis. However, with radiation therapy, autologous stem cell transplant, immunotherapy, and the differentiating agent isotretinoin, survival of high-risk neuroblastoma should approach 50% [**2**].

## Conclusion

**Learning points**

• Due to possible intra-tumoral hemorrhage, orbital neuroblastoma can reveal itself after minor head injury;

• In a presentation with acute/ rapid progressive proptosis, a tumoral etiology, such as neuroblastoma and rhabdomyosarcoma, should also be kept in mind;

• In case of an orbital tumor, a primary tumor should always be searched for elsewhere. 

**Acknowledgements**

The authors express their gratitude for the histopathology examination performed by Doctor Emil Botan.

**Informed consent**

The authors declare that the right to privacy of the patient was respected and informed consent was obtained.
